# Targeted Crystallization of Rare Earth Carbonate Polymorphs at Hydrothermal Conditions via Mineral Replacement Reactions

**DOI:** 10.1002/gch2.202200085

**Published:** 2022-09-19

**Authors:** Adrienn Maria Szucs, Melanie Maddin, Daniel Brien, Paul Christopher Guyett, Juan Diego Rodriguez‐Blanco

**Affiliations:** ^1^ Department of Geology School of Natural Sciences Trinity College Dublin Dublin 2 Ireland; ^2^ iCRAG Department of Geology School of Natural Sciences Trinity College Dublin Dublin 2 Ireland

**Keywords:** aragonite, bastnasite, dolomite, kozoite, lanthanite, rare earth carbonate

## Abstract

The interaction between rare earth element (REE)‐rich (La, Pr, Nd, Dy) aqueous solutions, dolomite (CaMg(CO_3_)_2_), and aragonite (CaCO_3_) at low temperature hydrothermal conditions (25–220 °C) is studied. The experiments result in the solvent‐mediated surface precipitation and subsequent pseudomorphic mineral replacement of the dolomite and aragonite seeds by newly formed REE‐carbonates. The host grains are replaced from periphery inward. The newly formed REE‐bearing carbonates in La‐, Pr‐, and Nd‐doped systems follow the crystallization sequence: lanthanite [REE_2_(CO_3_)_3_·8H_2_O] → kozoite [orthorhombic REECO_3_(OH)] → hydroxylbastnasite [hexagonal REECO_3_(OH)]. The interaction of Dy‐bearing solutions with dolomite results only in the crystallization of kozoite [orthorhombic DyCO_3_(OH)]. However, experiments with aragonite reveal a two‐step crystallization pathway: tengerite [Dy_2_(CO_3_)_3_·2‐3(H_2_O)] → kozoite [orthorhombic DyCO_3_(OH)]. The temperature, the dissolution rate of the host mineral, and the ionic radii of the REE^3+^ in question are found to control the kinetics of the replacement reaction, the polymorph selection, and the crystallization pathways toward bastnasite. The findings allow to gain a more in‐depth understanding of the formation REE‐bearing carbonates, particularly the mineral bastnasite, which is the main source of REEs for industry. This knowledge can be used to improve REE separation, exploration, exploitation methods, as well to produce carbonate minerals with tailored structures.

## Graphical abstract

The interaction of rare earth element (REE)‐bearing solutions and dolomite/aragonite results in solvent‐mediated surface precipitation and subsequent pseudomorphic mineral replacement of the host carbonate seeds by newly formed rare earth carbonates, following the crystallization sequence: lanthanite → kozoite → hydroxylbastnasite. The reaction is controlled by the temperature, ionic radii of the REE^3+^, and the solubility and dissolution rate of the host mineral.

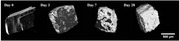

## 1 Introduction

Rare earth elements (REEs), also known as the lanthanides, are a set of elements that are playing a critical role in a sustainable and modern future as they are an essential part of many green energy technologies and electronic devices. REEs are usually divided into two subcategories: light rare earth elements (LREEs) are elements with atomic numbers 57–64, while atomic numbers 65–71 (and Y because of their similar chemical attributes) are defined as the heavy rare earth elements (HREEs). The U.S. Department of Energy categorized REEs in the short‐ and long‐terms as a function of supply risk and their importance in green energy technologies: Dy, Nd, Tb, Y, and Eu have been identified as critical raw materials in both short‐ and long‐term.^[^ ^1^ ^]^ La and Ce have been labelled as “near critical” while Pr have been described as “not critical” but the latter was included in their study as it can be a substitute for Nd in Nd–Fe–B, a permanent magnet used in many energy and military technologies.^[^ ^2^ ^]^

The wide applicability of REEs and their key role in modern devices and sustainable technologies resulted in an imbalance of supply and demand globally as the availability of REEs deposits with minable concentration are limited. According to the annual report of the U.S. Geological Survey (USGS),^[^ ^3^ ^]^ the global reserve of REEs rounds up to 120 Mtons, which supplied an estimated 280 000 tons in 2021; 40 000 higher than in 2020. Tracking back the USGS annual mineral commodity summaries on REEs, an increasing trend can be observed systematically through the last decade, which is driven by the constantly growing demand, pressuring REEs market for more.

Finding, extracting, and exploiting REEs reservoirs with minable concentration is alone challenging, but on the top of this, the deposits do not contain the idealized ratios to cover our REEs consumptions. For example, according to Alonso et al,^[^ ^4^ ^]^ a conventional mid‐size sedan car requires 0.45 kg REEs, which of ≈66% Nd, ≈18% Ce, ≈6% Dy, ≈2% La, and Pr, Sm, Eu, Gd, Tb also present with less than 1%. However, if we would like to be more sustainable and purchase a full hybrid vehicle with Li batteries, such a car would require more amount of REE (1.05 kg of REEs per car, where ≈73% Nd, ≈15% Dy, ≈8% Ce, ≈5% Tb, ≈3% and less than 1% of Pr, La, Eu, Gd, and Sm).^[^ ^4^ ^]^ Thus, the imbalance in supply and need stands and continuously rises as the population grows and as new innovative technologies/applications of REEs appear on the market.

The main source of REEs are carbonates, in particular, the minerals called bastnasite [(REE)CO_3_(OH)]; followed by phosphates, monazite [(REE)PO_4_] and xenotime [(Y,REE)PO_4_]. Nearly all REE carbonate deposits are dominated by the same four REEs: La, Ce, Pr, and Nd. The more valuable, “high‐risk” HREEs are still present in these reservoirs but typically with concentrations below 1%.^[^ ^5^, ^6^ ^]^ Thus, this concentration imbalance within REEs deposits results in further inconsistency of the REEs supply and need.

Alongside the natural limits of REE deposits, additional barriers are recognized in the REEs supply chain; the REEs market can be best described as volatile, and lacking transparency.^[^ ^7^ ^]^ Rapid changes in consumption patterns, speculation for market manipulation, connecting the global market with the suppliers and harmonizing environmental stewardship pose further challenges to the dynamics of the REEs supply‐demand chain.^[^ ^7^, ^8^ ^]^ China has been the main REE supplier since 1994,^[^ ^9^ ^]^ with their largest REE reserve at 44 Mtons. China exports the majority of the raw materials, including REEs, for green energy and modern technologies. In 2020–2021, over 69% of the world's solar panels,^[^ ^10^ ^]^ 79% of lithium‐ion batteries^[^ ^11^ ^]^ and also, over 60% of the total cellphones came from China. Through the years, countries have joined the REE competition, lining up behind China on the ranking list. Hence in 2021, Vietnam came as the second largest exporter with reserves of 22 Mtons, followed by Russia and Brazil ≈21 Mtons, while India came fourth in the list with ≈6.7 Mtons, Australia with ≈4 Mtons, the United States with ≈1.8 Mtons, Greenland as eighth with ≈1.5 Mtons, followed by Tanzania, Canada, South Africa, Thailand and other countries with reported supplies less than 1 Mton.^[^ ^3^ ^]^ As such, the majority of the REEs are supplied only by three countries, leaving the rest of the world dependent on the ruling of the leading suppliers.^[^ ^12^ ^]^ The world is completely dependent on REEs for modern technology: an estimated 350 kg of REEs (mostly Nd) is required by a 1.5 MW wind turbine, and a fully electric vehicle can include over 30 kg REEs in glass, electronics, batteries, and catalytic converters and each smartphone contains ≈50 mg of Nd and 10 mg of Pr.^[^ ^8^ ^]^ Thus, the plans of many countries to become carbon neutral, including China,^[^ ^13^ ^]^ in the next decades will require significant implications for green energy technologies and this will have a large impact on the REEs market, affecting the global exposure of China's dominance. Alternatively, e‐waste recycling could serve as a source for REEs in those countries that own no REEs reservoirs. However, REE recycling technologies are still in their infancy^[^ ^14^, ^15^, ^16^, ^17^, ^18^, ^19^ ^]^ as it only became economically viable when the market price of primary supplies became high due to the heavy demand.

The merging effects of the natural imbalance of REEs deposits and the dynamics of REEs economy result in the so‐called “rare earth crisis.” To find a solution, there need to be a clear understanding of the mechanisms controlling REEs concentrations in minerals, and also the factors affecting the mobility of REEs in the geosphere.

This research aims to gain a better understanding of the behavior of REEs in natural REE‐carbonate deposits. Our experimental methodology enables us to synthetize and characterize our own bastnasite samples instead of studying natural samples. This allows us to gain an in‐depth understanding of the crystallization process of rare‐earth carbonates under low‐hydrothermal and metasomatic conditions, which results in fundamental information on the individual role of REEs and the variables involved in the mineralization reactions.

In a previous study^[^ ^20^ ^]^ we investigated the crystallization kinetics of the interaction between rare‐earth‐bearing fluids and calcite crystals, finding out that bastnaesite forms via a multistep crystallization pathway. However, carbonate deposits consist of more than calcite only. Thus, to understand the mechanisms and pathways of REE carbonate crystallization, this study investigated the interaction of REE‐rich aqueous hydrothermal solutions with dolomite/aragonite crystals. La, Pr, Nd, and Dy elements were the subjects of our experiments because of the balance between the chemical similarities and dissimilarities of REEs. La represents the lightest rare earth element in the lanthanide series, Pr was selected as it is often used as substitute of Nd,^[^ ^2^ ^]^ Nd and Dy are in the high‐risk category.^[^ ^1^ ^]^ The purpose of this study is to mimic potential recrystallisation and replacement reactions involving dolomite and/or aragonite in REE deposits during hydrothermal and metasomatic processes. Hence the findings of this study provide primary knowledge on REE‐carbonates formation processes and crystallization kinetics, especially highlighting the influence of the individual REEs and the host carbonates, such as aragonite and dolomite in comparison with calcite. Thus, our results have a wide application as the information can be used to design and develop innovative materials and to improve REE separation, exploration, exploitation and recycling methods.

## 2 Results

The examination of solid samples obtained from the experiments revealed that the interaction between dolomite/aragonite seeds and rare earth bearing fluids resulted in newly formed phases as surface precipitates that partially or completely replaced the host minerals. The combination of binocular lens microscopy, X‐Ray diffraction (XRD), and scanning electron microscopy–energy dispersive X‐Ray spectroscopy (SEM‐EDX) techniques were used to identify and quantify these newly formed phases, and to derive the kinetics and mechanisms of rare earth carbonate formation.

### 2.1 Observation in Visible Light (Binocular Microscope)

The samples collected from La‐, Pr‐, Nd‐, and Dy‐bearing solutions at different times and temperatures were examined with a binocular microscope to gain preliminary information on the influence of time and temperature on the crystallization process.

The presence/absence and the volume of the precipitates occurring on the grains varied depending on the temperature and the duration of the reaction. The reaction took the most prolonged period to start at 21 °C with the first precipitate observed after 14 days in La‐, Pr‐, Nd systems on dolomite/aragonite; no precipitate was found during the observed period in Dy‐system. At 50 °C, a precipitate was visible after 1 day in the case of La‐doped dolomite/aragonite, Pr‐doped dolomite/aragonite and Nd‐doped aragonite. A slight delay (3 days) was observed in Nd‐induced dolomite system and no precipitate occurred during the observed period in the Dy‐experiment. At 80, 165, and 220 °C, a crystalline crust was visible on all REE‐doped dolomite/aragonite grains after 1 day. In addition, changes in the color and opacity of the dolomite and aragonite seeds were observed in all experiments where surface precipitation occurred. Dolomite grains were originally translucent, while aragonite seeds had a slight brownish color, but they became opaque by the end of all experiments. Dolomite seeds became light‐brown, light‐pink and green‐yellowish, while aragonite crystals turned into light‐pink and yellow, respectively (Figures S1 and S2, Supporting Information).

### 2.2 Powder X‐Ray Diffraction (XRD)

**Figure 1:**
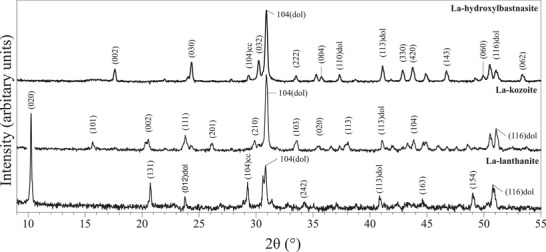
Powder XRD patterns of La‐lanthanite, La‐kozoite and La‐hydroxylbastnasite obtained as products of the experiments carried out with dolomite at 21, 50, and 220 °C, respectively.

**Figure 2:**
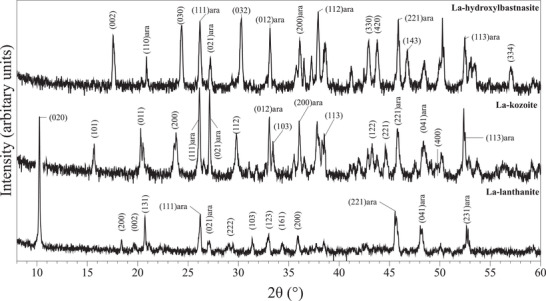
Powder XRD patterns of La‐lanthanite, La‐kozoite, and La‐hydroxylbastnasite obtained as products of the experiments carried out with aragonite at 21, 50, and 220 °C, respectively.

**Figure 3:**
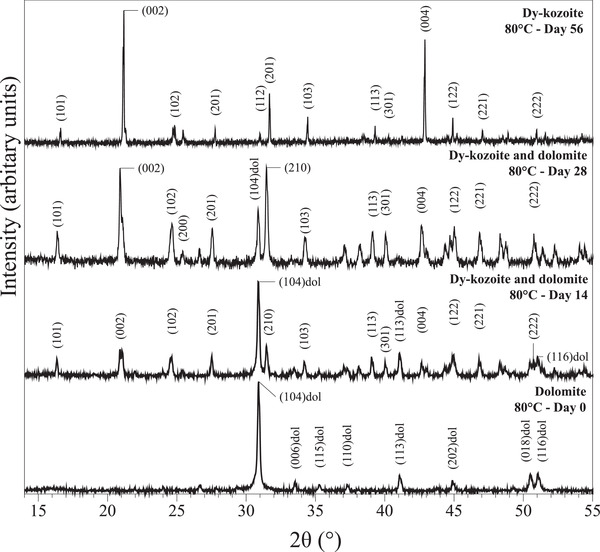
Powder XRD patterns showing the full replacement of dolomite by Dy‐kozoite at 80 °C within 56 days.

**Figure 4:**
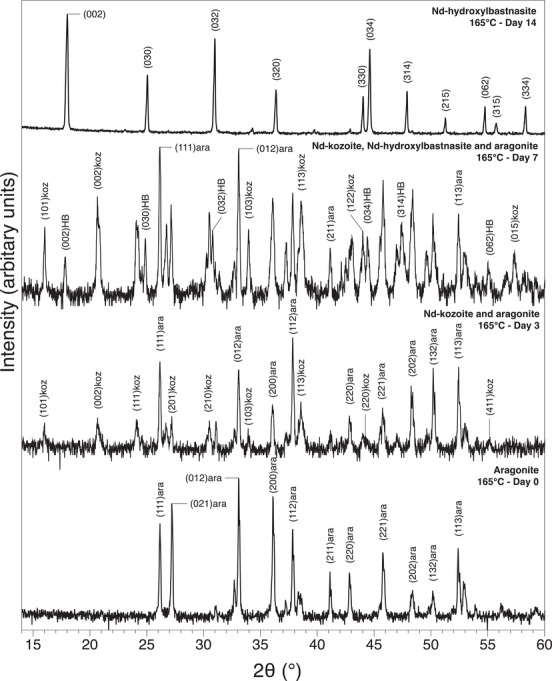
Powder XRD patterns showing the full replacement of aragonite by Nd‐hydroxylbastnasite via Nd‐kozoite at 165 °C within 14 days.

Characterization of samples with XRD allowed us to identify the newly formed secondary minerals. A series of different rare‐earth bearing carbonate minerals were detected: La‐, Pr‐, Nd‐lanthanite [(REE)_2_(CO)_3_·8H_2_O] (lan), Dy‐tengerite [(Dy_2_(CO_3_)_3_·2‐3(H_2_O)] (ten), La‐, Pr‐, Nd‐, Dy‐kozoite [(REE)CO_3_(OH)] (koz), La‐, Pr‐, Nd‐hydroxylbastnasite [(REE)CO_3_(OH)] (HB). Powder XRD patterns of these secondary minerals are presented on **Figures** **1**, **2**, **3**, **4** and Figure S3 (Supporting Information) and all experimental results are summarized in **Tables** **1**, **2**, **3**, **4**. Rietveld refinement revealed that the kinetics of crystallization of REE carbonates and the polymorph selection were temperature‐ and REE‐dependent. Regardless of the host mineral (dolomite/aragonite) and the composition of the newly formed crystals, all the seeds were fully replaced by crystalline rare earth carbonate minerals at temperatures ≥50 °C. The kinetics of these replacement reactions were temperature‐dependent: at 21 °C, the replacement reactions did not complete with dolomite being replaced at a maximum 15 wt% and aragonite at a maximum 10 wt% by the newly formed phase during the examined period of 56 days. However, at 50, 80, 165, and 220 °C, dolomite and aragonite grains were fully replaced after 56, 28, 14, and 3 days, respectively.

**Table 1:** Experimental conditions, identities, and morphologies of the solid rare earth carbonate phases formed during the interaction of dolomite or aragonite with lanthanum bearing aqueous solutions

Dolomite					Aragonite		
*T* [°C]	Time [days]	%Phase consumed	Phase formed	Morphology	%Phase consumed	Phase formed	Morphology
21	14	4	Lan	Thin platy crystals	5	Lan	Thin platy crystals
	28	9			7		
	42	11			8		
	56	15			10		
	*n* = 1.0 *k* (×10^−6^) = 0.03 s^−1^				*n* = 0.5 *k* (×10^−6^) = 0.002 s^−1^		
50	1	3	Lan	Lan: Thin platy crystals Koz: Small prisms, Elongated prisms	42	Lan	Lan: Thin platy crystals Koz: Small prisms, Elongated prisms
	3	5			58	29% Lan + 29% Koz	
	7	33	3% Lan + 30% Koz		79	42% Lan + 37% Koz	
	14	50	25% Lan + 25% Koz		88	59% Lan + 29% Koz	
	28	80	Koz		96	8% Lan + 88% Koz	
	56	100			100	4% Lan +96% Koz	
	*n* = 1.5 *k* (×10^−6^) = 0.80 s^−1^				*n* = 0.8 *k* (×10^−6^) = 3.46 s^−1^		
80	1	19	6% Lan + 13% Koz	Lan: Thin platy crystals Koz: Small prisms, Elongated prisms	44	Koz	Small prisms, Elongated prisms
	7	25	17% Lan + 8% Koz		64		
	14	57	21% Lan + 36% Koz		84		
	28	100	Koz		100		
	*n* = 1.1 *k* (×10^−6^) = 1.54 s^−1^				*n* = 0.8 *k* (×10^−6^) = 3.72 s^−1^		
165	3	33	HB	Triangular prisms	31	31% Koz	Triangular prisms
	7	80			69	11% Koz + 58% HB	
	14	100			100	HB	
	*n* = 2.4 *k* (×10^−6^) = 2.43 s^−1^				*n* = 2.3 *k* (×10^−6^) = 2.34 s^−1^		
220	1	91	HB	Triangular prisms	72	HB	Triangular prisms
	3	100			100		
	*n* = 1.7 *k* (×10^−6^) = 19.22 s^−1^				*n* = 2.3 *k* (×10^−6^) = 12.84 s^−1^		

**Table 2:** Experimental conditions, identities, and morphologies of the solid rare earth carbonate phases formed during the interaction of dolomite or aragonite with praseodymium bearing aqueous solutions

Dolomite					Aragonite		
*T* [°C]	Time [days]	%Phase consumed	Phase formed	Morphology	%Phase consumed	Phase formed	Morphology
21	14	<2	Lan <1% + Koz <1%	Lan: Thin platy crystals Koz: Small prisms, Elongated prisms	14	Lan	Thin platy crystals
	28	5	1% Lan + 4% Koz		21		
	42	8	2% Lan + 6% Koz		23		
	56	10	3% Lan + 7% Koz		24		
	*n* = 1.2 *k* (×10^−6^) = 0.03 s^−1^				*n* = 0.4 *k* (×10^−6^) = 0.01 s^−1^		
50	1	20	Koz	Small prisms, Elongated prisms	24	3% Lan + 17% Koz	Lan: Thin platy crystals Koz: Small prisms, Elongated prisms
	3	25			33	4% Lan + 29% Koz	
	7	50			43	2% Lan + 41% Koz	
	14	60			57	Koz	
	28	95			89		
	56	100			100		
	*n* = 1.0 *k* (×10^−6^) = 1.47 s^−1^				*n* = 0.9 *k* (×10^−6^) = 1.47 s^−1^		
80	1	22	Koz	Small prisms, Elongated prisms	47	Koz	Small prisms, Elongated prisms
	7	31			81		
	14	82			91		
	28	100			100		
	*n* = 1.1 *k* (×10^−6^) = 1.97 s^−1^				*n* = 0.8 *k* (×10^−6^) = 4.78 s^−1^		
165	3	12	4% Koz + 8% HB	Koz: Small prisms, Elongated prisms HB: Triangular prisms	50	47% Koz + 3% HB	Koz: Small prisms, Elongated prisms HB: Triangular prisms
	7	80	HB		87	77% Koz + 10% HB	
	14	100			100	HB	
	*n* = 2.4 *k* (×10^−6^) = 2.43 s^−1^				*n* = 2.0 *k* (×10^−6^) = 2.91 s^−1^		
220	1	78	51% Koz + 27% HB	Koz: Small prisms, Elongated prisms HB: Triangular prisms	50	Koz	Koz: Small prisms, Elongated prisms HB: Triangular prisms
	3	100	HB		100	34% Koz + 66% HB	
	*n* = 2.2 *k* (×10^−6^) = 14.03 s^−1^				*n* = 2.9 *k* (×10^−6^) = 10.18 s^−1^		

Additionally, a trend was observed in the kinetics of replacement of dolomite/aragonite by the secondary phases: La‐bearing experiments were always the first to initiate crystallization on the surface of the host mineral, followed by Pr‐ and later, by Nd‐bearing experiments. By contrast, the onset of the crystallization and kinetics of replacement were found to be the slowest in the Dy‐bearing system, with no replacement reaction occurring below 80 °C (Table 4).

Lanthanite was generally present at low temperatures, 21–50 °C. While at 21 °C, it remained stable for the full duration of the experiment, at 50 °C, it slowly transformed into kozoite between 14 and 28 days. This mineral was never detected in the Dy‐system, but instead, another hydrated phase, Dy‐tengerite, was observed in the experiments with aragonite at 80 °C.

Kozoite occurred at all the examined temperatures, from 21 to 220 °C. Up until 80 °C, kozoite remained stable until the end of the experiments. At higher temperatures, 165–220 °C, kozoite eventually transformed into hydroxylbastnasite, with the only exception of Dy‐doped experiments, where no Dy‐hydroxylbastnasite was detected and Dy‐kozoite was found to be the final stable mineral. REE‐hydroxylbasnasite crystallized as the final product of the experiments at 165–220 °C, thus, it was the most thermodynamically stable phase and the end product of this crystallization sequence.

### 2.3 Scanning Electron Microscopy (SEM)

**Figure 5:**
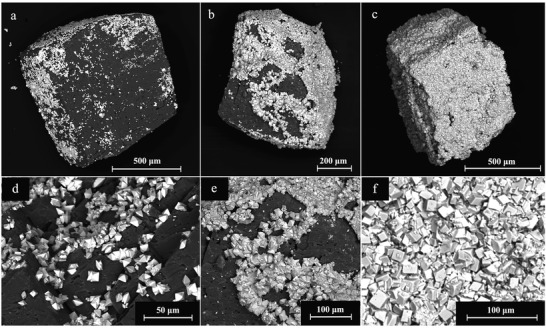
SEM photomicrographs of the surface of dolomite grains interacted with Dy‐bearing solutions at 80 °C. a) After 3 days of reaction, some crystals of Dy‐kozoite are scattered on the surface of calcite. b) Dy‐ kozoite crystals almost fully cover the available surface after 7 days. c) The full dolomite grain is fully replaced by Dy‐kozoite after 56 days. Detail of Dy‐kozoite after d) 3 days, e) 7 days and, f) 56 days.

SEM images showed the precipitate on the surface of the dolomite and aragonite grains. A representative example is showed in **Figure** **5**, presenting the surface of a typical dolomite grain after 3 (Figure 5a), 7 (Figure 5b), and 56 days (Figure 5c) of reaction with Dy‐bearing parent solution at 80 °C. Clear dissolution signs occurred after a short reaction time and small (≈20 µm) individual crystals started to cover the host grain (Figure 5a,d). Over time, this new precipitate phase continued to extend on the surface (Figure 5b,e), eventually covering the host seed (Figure 5c). The final result was a completed pseudomorphic replacement of the dolomite grain in which the original morphology and dimensions of the host crystal were preserved (Figure 5c). The host mineral was fully replaced by the new REE carbonate phase, a replacement also confirmed by XRD (Figure 3). SEM imaging also allowed to study the effects of temperature, rare‐earth element chemistry, and host mineral on the surface precipitation process, as well as the morphologies of the secondary minerals. The chemistry of these secondary phases was also confirmed by energy dispersive spectroscopy (EDS) microanalysis carried out on selected crystals. The content of Ca and Mg of the newly formed phases was overall very low (atom % ≤ 1.5) or below the detection limit of the instrument (Tables S1 and S2, Supporting Information) and tended to be lower in the samples obtained at higher temperatures.

**Figure 6:**
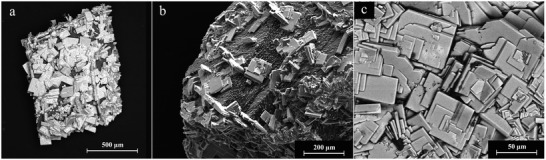
SEM photomicrographs showing: a) The surface of dolomite almost fully covered by La‐lanthanite after 56 days of reaction at 21 °C. b) SEM‐BSE image of La‐lanthanite crystals on the surface of dolomite. c) Morphology of La‐lanthanite single crystals growing on the surface of aragonite at 21 °C.

**Figure 7:**
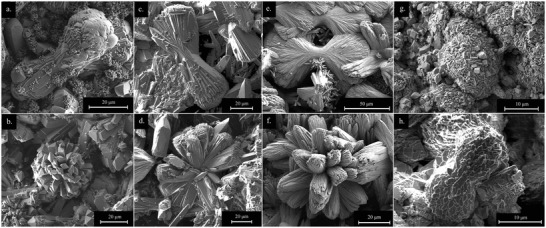
SEM micrographs of spherulitic morphologies of a,b) La‐kozoite, c,d) Pr‐kozoite, e,f) Nd‐kozoite, g) Dy‐kozoite, and h) Dy‐tengerite.

**Figure 8:**
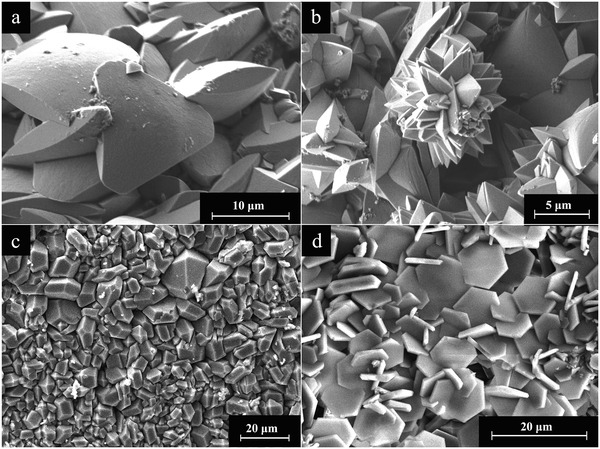
SEM images showing a) morphology of La‐hydroxylbastnasite single crystals; b) spherulites of Nd‐hydroxylbastnasite; c) La‐hydroxylbastnasite replacing dolomite at 220 °C; d) hexagonal crystals of Pr‐hydroxylbastnasite formed at 220 °C.

Regarding the morphology, the REE‐bearing carbonate phases showed similar morphologies with some variations (Figure S4, Supporting Information). La‐, Pr‐, and Nd‐lanthanite developed crystals with sizes ranging ≈20–100 µm, and thin platy morphologies both on dolomite (**Figure** **6**a,b) and aragonite (Figure 6c). La‐, Pr‐, and Nd‐kozoite evolved as elongated prisms (≈100 µm), forming spindle‐like shapes and, sometimes, spherulitic morphologies consisting of crystalline nanoaggregates (**Figure** **7**). Dy‐kozoite significantly differed from the rest, by forming prisms with size ranging ≈5–25 µm, and sometimes spherical aggregates (Figures 5 and 7d and Figure S5, Supporting Information). Dy‐tengerite was built by aggregates of flat‐bladed crystals with size of ≈1–2 µm, which also had a tendency for aggregating forming elongated spherules (Figure 7h). Finally, REE‐hydroxylbasnasite crystallized as triangular prisms with sizes of ≈10 µm after short reaction times (≈1–3 days) (**Figure** **8**a), which later evolved into slightly larger (≈20–40 µm) rectangular prisms (Figure 8c). Some REE‐hydroxylbasnasite also formed spherulitic aggregates but this was less common (Figure 8b). Also, at 220 °C, only in the case of Pr‐dolomite and Dy‐dolomite, hexagonal morphologies were also observed (Figure 8d and Figure S5, Supporting Information).

### 2.4 Quantitative Kinetic Studies

**Figure 9:**
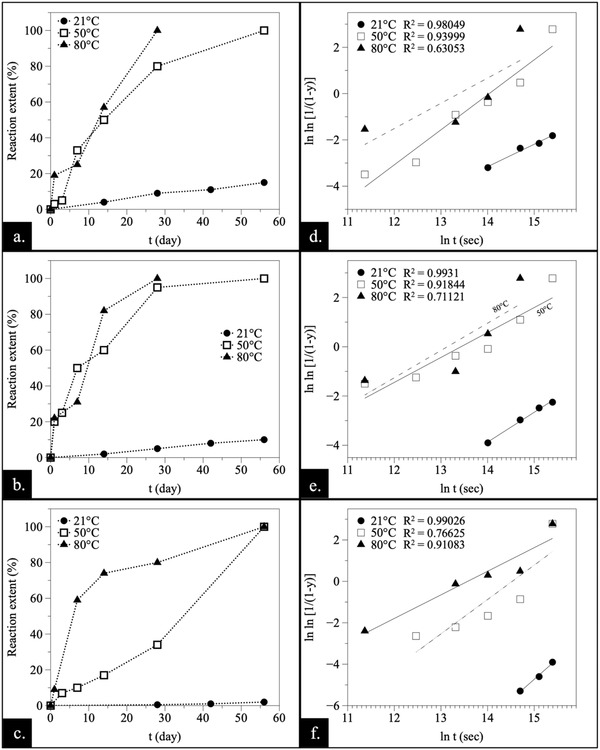
Reaction extents plotted against time a–c) and the corresponding Avrami plots d–f) for dolomite experiments with a,d) La, b,e) Pr, and c,f) Nd showing the effect of the temperature.

**Figure 10:**
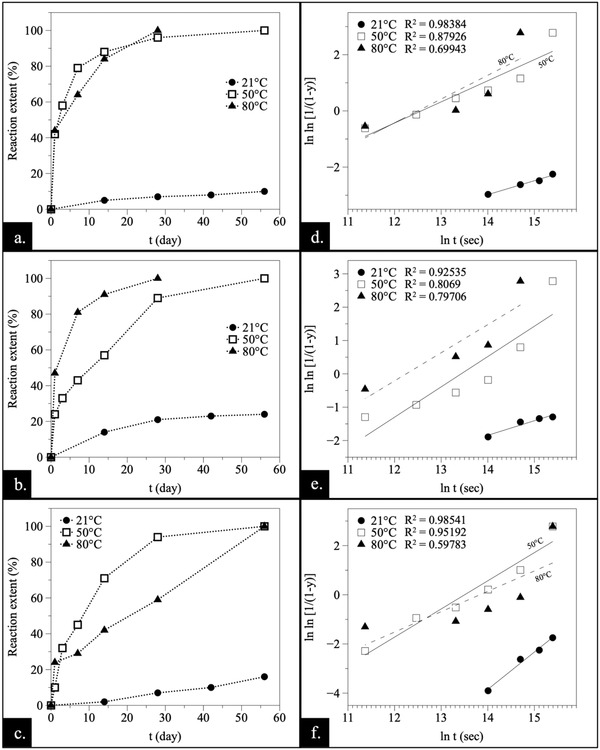
Reaction extents plotted against time a–c) and the corresponding Avrami plots d–f) for aragonite experiments with a,d) La, b,e) Pr, and c,f) Nd showing the effect of the temperature.

Graphing the quantitative values of Table 1, 2, 3, 4, a temperature‐dependent induction period was observed in the lower temperature experiments (21–80 °C) (**Figures** **9**a–c and **10**a–c). At 21 °C, the onset of the crystallization took place after weeks, while at higher temperatures (220 °C) the newly formed phases were detected within 24 h. Also, the lower the temperature, the longer the time that was needed to complete the replacement reactions. In fact, the higher temperature (165–220 °C) experiments have not been included as part of Figures 9 and 10 because replacement reactions were rapidly completed, so following them in‐depth with our experimental methodology would have been too challenging, and beyond the scope of this study. Also, the REE chemistry had an impact on the required induction time. Typically, the onset of the crystallization in systems with heavier rare earth elements took place after longer induction times (Figure S6, Supporting Information).

**Table 3:** Experimental conditions, identities, and morphologies of the solid rare earth carbonate phases formed during the interaction of dolomite or aragonite with neodymium bearing aqueous solutions

Dolomite					Aragonite		
*T* [°C]	Time [days]	%Phase consumed	Phase formed	Morphology	%Phase consumed	Phase formed	Morphology
21	14	0	Koz	Small prisms, Elongated prisms	2	<1% Lan + 1% Koz	Lan: Thin platy crystals Koz: Small prisms, Elongated prisms
	28	<1			7	3% Lan + 4% Koz	
	42	1			10	6% Lan + 4% Koz	
	56	2			16	10% Lan + 6% Koz	
	*n* = 2.0 *k* (×10^−6^) = 0.03 s^−1^				*n* = 1.5 *k* (×10^−6^) = 0.07 s^−1^		
50	1	0	Koz	Small prisms, Elongated prisms	10	Koz	Small prisms, Elongated prisms
	3	7			32		
	7	10			45		
	14	17			71		
	28	34			94		
	56	100			100		
	*n* = 1.7 *k* (×10^−6^) = 0.49 s^−1^				*n* = 1.1 *k* (×10^−6^) = 1.37 s^−1^		
80	1	9	Koz	Small prisms, Elongated prisms	24	Koz	Small prisms, Elongated prisms
	7	59			29		
	14	74			42		
	28	80			59		
	56	100			100		
	*n* = 1.1 *k* (×10^−6^) = 1.28 s^−1^				*n* = 0.8 *k* (×10^−6^) = 0.98 s^−1^		
165	3	14	Koz	Koz: Small prisms, Elongated prisms HB: Triangular prisms	61	Koz	Koz: Small prisms, Elongated prisms HB: Triangular prisms
	7	19	12% Koz + 7% HB		74	28% Koz + 46% HB	
	14	96	89% Koz + 7% HB		100	HB	
	28	100	7% Koz + 93% HB				
	*n* = 2.2 *k* (×10^−6^) = 1.29 s^−1^				*n* = 1.8 *k* (×10^−6^) = 3.05 s^−1^		
220	1	52	12% Koz + 40% HB	Koz: Small prisms, Elongated prisms HB: Triangular prisms	50	Koz	Koz: Small prisms, Elongated prisms HB: Triangular prisms
	3	100	HB		100	83% Koz + 17% HB	
	*n* = 2.9 *k* (×10^−6^) = 10.18 s^−1^				*n* = 2.9 *k* (×10^−6^) = 10.18 s^−1^		

**Table 4:** Experimental conditions, identities, and morphologies of the solid rare earth carbonate phases formed during the interaction of dolomite or aragonite with dysprosium bearing aqueous solutions

Dolomite					Aragonite		
*T* [°C]	Time [days]	%Phase consumed	Phase formed	Morphology	%Phase consumed	Phase formed	Morphology
21	14	0	–	–	0	–	–
	28						
	42						
	56						
50	1	0	–	–	0	–	–
	3						
	7						
	14						
	28						
	56						
80	1	0	Koz	Small prisms, Elongated prisms	0	–	Koz: Small prisms, Elongated prisms Ten: Aggregates of flat‐bladed crystals
	7	12			57	40% Koz + 17% Ten	
	14	23			71	50% Koz + 21% Ten	
	28	30			96	91% Koz + 5% Ten	
	56	100			100	Koz	
	*n* = 12.1 *k* (×10^−6^) = 0.48 s^−1^				*n* = 1.4 *k* (×10^−6^) = 1.19 s^−1^		
165	3	27	Koz	Small prisms, Elongated prisms	64	Koz	Small prisms, Elongated prisms
	7	33			76		
	14	50			100		
	28	100					
	*n* = 1.6 *k* (×10^−6^) = 1.28 s^−1^				*n* = 1.7 *k* (×10^−6^) = 3.18 s^−1^		
220	1	24	Koz	Small prisms, Elongated prisms	79	Koz	Small prisms, Elongated prisms
	3	100			100		
	*n* = 3.7 *k* (×10^−6^) = 8.2 s^−1^				*n* = 2.1 *k* (×10^−6^) = 14.27 s^−1^		

The rate of transformation, determined by the *Avrami* equation and plotting − ln ln (1 − *y*) against ln *t* (Figures 9d–f and 10d–f), allowed us to gain a better understanding of the reaction mechanisms. The *n* and *k* values of the experiments have been included in Table 1, 2, 3, 4. The estimated *k* values also provided primary information on the kinetics of the crystallization process. It is important to highlight that the *n* and *k* values have been estimated but this must be handled carefully as there are limited data points available to establish a precise value for these experiments especially for those at 165 and 220 °C.

Hydrogeochemical modeling confirmed that during the interaction of aragonite and dolomite with REE‐bearing solutions, the aqueous solution became highly supersaturated with respect to the REE‐carbonates (lanthanite, kozoite and hydroxylbastnasite) in all experiments. For example, when the REE‐bearing solution equilibrated with host mineral at ambient temperature, it was already supersaturated with respect to La‐lanthanite [SI > 8.58 (dolomite); SI > 9.23(aragonite)], La‐hydroxylbastnasite [SI > 8.40 (dolomite); SI > 8.77 (aragonite)], similarly at 165 °C for La‐lanthanite [SI > 1.14 (dolomite); SI > 2.50 (aragonite)], La‐hydroxylbastnasite [SI > 5.92 (dolomite); SI > 6.58 (aragonite)]. It must be noted that there is a lack of thermodynamic data about the stabilities of the aqueous complexes and solubility products must be mentioned, as a limiting factor for carrying out the calculations (e.g., no modelling is possible with Dy‐kozoite as no solubility product has been determined).

## 3 Discussion

Our experiments result in the formation of a surface precipitate on the host grains consisting of different REE carbonates. Once the host minerals are placed in contact with the starting aqueous solutions, Ca^2+^, Mg^2+^, and CO_3_^2−^ are released from the surface of dolomite/aragonite. As the concentration of Ca^2+^ and CO_3_^2−^ increases in the liquid, especially close to the surface of the grains, the CO_3_^2−^ ions react with the dissolved REE^3+^ ions in the aqueous solution, resulting in the heterogeneous nucleation and growth of different REE‐carbonate polymorphs on the surface of the host seeds. The combination of XRD and SEM has revealed a solution‐mediated pseudomorphic replacement of the host grain(s) by REE‐bearing phases. Concurrently, the newly formed phases follow a temperature‐dependent transformation from lanthanite (or tengerite, only in the Dy system), to kozoite and hydroxylbasnasite. This crystallization mechanism is consistent with the previously investigated REE‐bearing fluid and calcite interaction by Szucs et al.^[^ ^20^ ^]^

Epitaxial overgrowth in the experiments carried out with dolomite/aragonite were expected as it was observed on calcite, reported in a previous work by Szucs et al.^[^ ^20^ ^]^ However, we have not observed any epitaxial overgrowth in dolomite/aragonite‐hosted experiments at any REE‐bearing system at any temperatures. We cannot exclude the possibility that an epitaxial overgrowth occurred in the earliest stages of the replacement reaction, a period of time in which we were unable to follow the reaction because of the fast kinetics of the replacement reaction.

**Figure 11:**
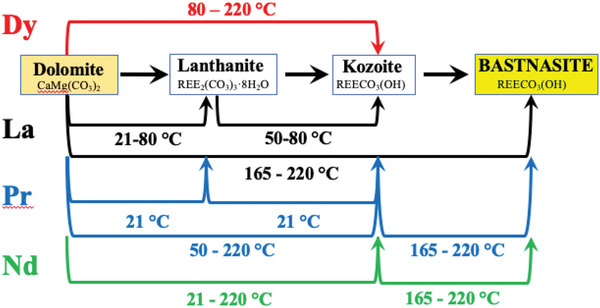
Reaction pathways toward bastnasite observed during the replacement of dolomite by rare earth carbonates. Lines correspond to observed reactions in our experiments and each line shows the temperature at which the reaction takes place.

In general, our experiments evolved to reveal a crystallization sequence beginning with lanthanite, followed by kozoite and ending with hydroxylbastnasite. Exception to this is in the Dy‐dolomite experiments where Dy‐kozoite is the final stable phase (**Figure** **11**). This phenomenon was also observed by Szucs et al.,^[^ ^20^ ^]^ with calcite grains; Dy‐kozoite did not transform into Dy‐hydroxylbastnasite even after one months at 160 °C while La‐ and Nd‐doped experiments replaced the full calcite grain within one day. This has been observed in crystallization from solution experiments by Vallina et al.,^[^ ^21^ ^]^ showing that kozoite‐Dy does not transform to any other phase. Dy‐bastnasite at this P‐T conditions cannot form as the size of the Dy^3+^ ion is too small. In nature, bastnasite contains larger REE ions (from La^3+^ to Nd^3+^) while Dy^3+^ and smaller REEs (heavier REEs) only enter as impurities in low concentrations.^[^ ^22^, ^23^ ^]^

**Figure 12:**
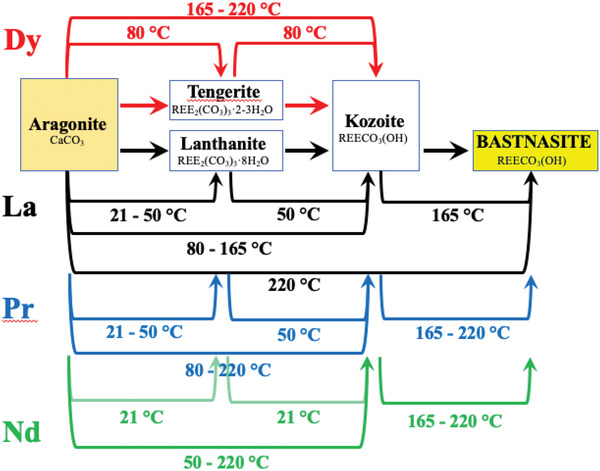
Reaction pathways toward bastnasite observed during the replacement of aragonite by rare earth carbonates. Lines correspond to observed reactions in our experiments and each line shows the temperature at which the reaction takes place.

Experiments with aragonite (**Figure** **12**) showed a crystallization sequence similar to dolomite, when comparing to La, Pr‐, and Nd‐systems. However, the first newly formed phase in the experiments with aragonite in Dy‐bearing solutions was Dy‐tengerite, which transformed to Dy‐kozoite after more than a month of reaction, at 80 °C. Dy‐tengerite was only observed in aragonite experiments but never detected in association with dolomite. This REE‐bearing polymorph selection is dependent on the temperature, ionic radii of the REE involved, and the solubility and dissolution rate of the host mineral. The effects of these three variables are discussed in detail below:

### 3.1 The Importance of Solubility and Dissolution Rate of the Host Mineral

In all experiments, the host grains were replaced periphery inward (centripetal replacement), which occurs because the newly formed phase is less soluble than the host mineral at the observed P–T range. There is a limited amount of research on the solubility products of REE‐carbonates and their polymorphs, but it is well known that divalent carbonates (calcite, aragonite, and dolomite) are several orders of magnitude more soluble than La‐ and Nd‐lanthanite, Nd‐kozoite and, La‐, and Nd‐hydroxylbasnasite.^[^ ^24^, ^25^, ^26^ ^]^ It should be mentioned that we should expect that these REE‐carbonate phases have retrograde solubilities and this would influence the coupling between dissolution and precipitation in our systems. However, the existing bibliographic information on REE‐carbonate solubility products is insufficient to confirm this and we should also expect inconsistencies in the calculations of these solubility products due to the presence of REE impurities in the solids as well as variations in the particle size of the solids used for the solubility product determinations. In our experimental system, the key factor to explain the replacement behavior is the difference in solubility between the Ca–Mg carbonates and the REE‐carbonates, which are so large that the retrograde solubility effect of the REE‐carbonates becomes negligible.

Also, we must elaborate on the relationship between dissolution of the host mineral and the porosity of the newly formed REE‐carbonate. The molar volumes of kozoite (e.g., Nd‐kozoite = 46.39 cm^3^ mol^−1^) and bastnasite (e.g., Nd‐hydroxylbastnasite = 43.46 cm^3^ mol^−1^) are similar to dolomite (64.12 cm^3^ mol^−1^) and aragonite (34.17 cm^3^ mol^−1^). A similarity in molar volumes can often be translated in partial equilibrium situations (e.g., Helgeson, 1968;^[^ ^27^ ^]^ Rodriguez et al., 2008^[^ ^28^ ^]^) when the reactive solid (aragonite/dolomite) becomes isolated from the aqueous solution by a crust of secondary solids that maintain equilibrium with the aqueous phase. Despite the differences in molar volumes, a partial equilibrium situation does not seem to have happened in our experiments as full replacement was observed in most cases. We suggest that the full replacement reaction we observed could be a consequence of having used a small enough host grain size (0.5–1.0 mm), and we cannot exclude the possibility of reaching a partial equilibrium situation with larger (e.g., >2 mm) host grains. In fact, we cannot discard a certain influence of the forming crust on the dissolution rate of the host minerals and on their transformation rate. This situation would especially affect the latest stages of the replacement reaction, as the crust becomes thicker, and would contribute to errors in the Avrami plots. On the other side, we also expect the development of porosity in many of our experiments because the formation of crystals with defects and discontinuities usually happens as a consequence of rapid crystal growth (including spherulitic growth processes). However, studying the porosity of these newly formed REE‐carbonates is beyond the scope of this study with this experimental set‐up. The only experiments where no full replacement of the host grain took place were carried out at ambient conditions (25 °C) and resulted in the surface precipitation of lanthanite. The molar volume of lanthanite (e.g., La‐lanthanite = 220.32 cm^3^ mol^−1^) is much larger compared to dolomite or aragonite, so these would the most favorable cases for the partial equilibrium situation to occur. Although very feasible, it is difficult to determine if this would happen because the kinetics of lanthanite growth is very slow and the surfaces of the host minerals were not fully covered even after long (145 days) reaction times.

In all the lower temperature experiments (≤80 °C), aragonite crystals were replaced by REE‐bearing minerals at a faster rate than dolomite (Tables 1, 2, 3, 4). At the highest temperatures, it was not possible to obtain good‐quality time‐resolved data with our experimental method. The difference in the reaction extents of aragonite and dolomite experiments can be explained by the solubility and dissolution rates of the host minerals. Aragonite (log *K*_ara_ = −8.34)^[^ ^29^ ^]^ is more soluble than dolomite (log *K*_dol_ = −18.14).^[^ ^29^ ^]^ In addition, the dissolution rates of dolomite and aragonite, determined by Chou et al.^[^ ^30^ ^]^ show that aragonite (log *R*_ara_ = −9.2 mol cm^−2^ s^−1^) dissolves nearly 10 times faster compared to dolomite (log *R*_dol_ = −10.1 mol cm^−2^ s^−1^) at pH 5.1, which was the pH of our aqueous solution at the beginning of the reaction. It is worth mentioning that Busenberg and Plummer^[^ ^31^ ^]^ had estimated dolomite dissolution a few years before, but we have used data determined by Chou et al.,^[^ ^30^ ^]^ who took into account the effect of atmospheric P_CO2_. Besides, we also must bear in mind that the carbonate minerals used by Chou et al.^[^ ^30^ ^]^ and Busenberg et al. ^[^ ^31^ ^]^ were sourced from nature, thus their determined values may differ slightly due to the presence of trace impurities. The difference in the dissolution rates of aragonite and dolomite means that only after a few minutes of reaction the aqueous solutions became supersaturated with respect to REE‐bearing carbonates, but these supersaturation levels in the aragonite experiments were reached ≈10 times faster compared to dolomite. With a higher dissolution rate, aragonite can release more carbonate ions into the aqueous solution at the early stage of the reaction, thus high supersaturation levels required for the onset of nucleation can occur faster than in the dolomite experiments. The dissolution of a few monolayers of the host mineral's surface sufficiently increases the supersaturation levels, forming a fluid boundary layer near‐surface of the host grain, which promotes the formation of a surface precipitate. Also, the surface precipitation of the new REE‐carbonate phase would decrease the concentration of carbonate ions in the aqueous solution, further enhancing the dissolution rate of the host mineral and increasing the growth rate of the overgrowth. PHREEQC calculations suggest that the REE‐carbonate formation is likely to occur at the early stages of the host mineral dissolution. Also, the development of the spherulitic growth requires high supersaturation levels (SI > 2–3), and the calculated saturation indices in both the aragonite and dolomite experiments are high enough to promote this growth mechanism, an indication of the importance of the kinetics of dissolution of the host minerals. These high supersaturation levels, near the surface of the host are translated into fast crystal growth velocities, which result in crystal aggregates containing growth defects or spherulitic morphologies. In our experiments, spherulitic growth occurred at temperatures >80 °C (Figures 7 and 8b) regardless of the chemistry of the REE involved. This also explains the morphology of the newly formed phases, which tend to be more irregular and have smaller crystal sizes on aragonite, compared to dolomite (or calcite^[^ ^20^ ^]^). Higher supersaturation levels at the early stages of the reaction could also explain the crystallization of primary Dy‐tengerite in the aragonite experiments at 80 °C, which would require higher supersaturation levels to form,^[^ ^21^ ^]^ compared to the more insoluble Dy‐kozoite.

As the reaction proceeds, the pH drifts to more basic values and the dissolution rate of the host mineral became controlled by multiple factors, i.e., the growth kinetics of REE‐carbonates, the available surface area of the host mineral, the initial concentration of REE^3+^ in the aqueous solution and temperature.

### 3.2 The Combined Effects of Temperature and REE Ionic Radii

Temperature affects the reaction by governing the intensity of the kinetic energy in the system. In general, the effect of the temperature follows an increasing trend: the higher the temperature is, the faster the reaction becomes. This trend is highlighted on Figures 9a–c and 10a–c, where the reaction extents of La, Pr and Nd experiments at 21, 50, and 80 °C have been plotted against time. Above this temperature, the reaction is completed so rapidly that it is not possible to obtain enough data points for defining an actual curve. Similarly, Dy experiments have not been plotted at the higher temperatures for the same reason. Also, both 21 and 50 °C Dy‐experiments were omitted as no reaction was initiated at these temperatures during the observed period. The data analysis through *Avrami* equation (Figures 9d–f and 10d–f) reveals transformation rates showing close to parallel lines with similar *n* values, indicating no evident change in the reaction mechanisms of the replacement process. However, these results must be treated with sufficient care as there is limited amount of data points available to strongly support Avrami plotting. The given *R* ^2^ values show how well the linear regression fits; the dash line indicates the weak fitting (Figures 9d–f and 10d–f). To conclude the level of validity of this information, standard error values of each slope were provided in Table S3 (Supporting Information).

The temperature governs the crystallization kinetics by providing enough energy for the desolvation of the REE^3+^ ions. To initiate the nucleation of a crystal, the REE^3+^ ions in solution need to lose their hydration shells; the higher the ionic potential of an ion, the higher the energy required to remove the solvation shell. Similar behavior is known to happen with the divalent cations Ca^2+^ and Mg^2+^ during the formation of minerals like calcite, monohydrocalcite, and dolomite.^[^ ^32^, ^33^, ^34^, ^35^ ^]^ In the case of the REE^3+^, each ion has a different ionic potential depending on its ionic radius and therefore different energy levels are required to overcome the kinetic barrier needed to dehydrate their solvation shells. The higher the temperature, the higher the available energy to remove the solvation shell of the ions in the solution.^[^ ^21^, ^36^, ^37^ ^]^ The reaction can thus proceed faster at higher temperatures, reducing the required time to complete the reaction process. The ionic potentials of La^3+^, Pr^3+^ Nd^3+^, and Dy^3+^ are 2.60, 2.75, 2.77, and 3.03 Å^−1^. La^3+^ requires the lowest energy to remove the solvation shell, followed by Pr^3+^ and Nd^3+^, while Dy^3+^ requires the highest energy. Therefore, Dy^3+^ requires the highest temperature (or longer time) to dehydrate and incorporate into the newly forming crystalline structures, compared to REEs with lower ionic potentials. This explains why the onset of crystallization took longer for heavier REEs compared to lighter REEs. Also, it explains the fact that no replacement reaction in the Dy‐system took place in 56 days at 21 and 50 °C; at these temperatures, Dy^3+^ requires more time to lose the solvation shell and initiate the crystallization. A similar behavior was observed in calcite (Szucs et al., 2021) which is also consistent with observations of REE‐carbonate crystallization from solution.^[^ ^36^, ^38^ ^]^ In all the cases, this behavior is consistent with the ionic potential of the REE^3+^ ions and can be explained by the strength of the cations to desolvate their hydration shells by increasing the induction times of crystallization or requiring higher T to crystallize. Fedorov et al.,^[^ ^39^ ^]^ observed that phase transitions in REE‐carbonates are also dependent on the strengths of their hydration shells: the heavier REEs ions require temperatures >700 °C to release strongly bonded residual water, while this release occur at lower temperatures for the lighter REEs.

Dy experiments are also great examples to observe the effect of the slow kinetics of cation desolvation derived from the higher ionic potential of the REE in question at the lower temperatures (≤80 °C). The ionic potential of the REE^3+^ can contribute to the supersaturation level prior to nucleation: Higher ionic potentials of the REE^3+^ allow more time for the host mineral to dissolve, releasing more Ca^2+^, Mg^2+^, and CO_3_^2−^ ions into the aqueous solution, increasing the supersaturation levels prior to nucleation and hence the driving force of the crystallization. In agreement, our data shows that Dy experiments required more time to initiate crystallization and that Dy‐kozoite crystals tend to be smaller (5–10 µm) (Figures 5d and 7d) compared to kozoite from the La, Pr, or Nd experiments (>50 µm) (Figure 7a–c), suggesting that the former crystallized at higher supersaturation levels compared to the later.

The crystallization of the REE carbonates formed via a single or multiple metastable phases follows the sequence from more soluble to less soluble stages. This is a well‐known phenomenon, under the name of “Ostwald's Rule of Stages,”^[^ ^40^ ^]^ observed in many mineral systems (e.g., Dae et al., Bots et al., or Van Driessche et al.^[^ ^41^, ^42^, ^43^ ^]^). Our experiments showed that hydroxylbasnasite forms via two metastable phases: lanthanite → kozoite → hydroxylbastnasite. However, specific REEs favored particular phases (Tables 1, 2, 3, 4 and Figures 11 and 12). In the case of La‐ and Pr‐bearing experiments, the full crystallization sequence occurred in both dolomite and aragonite systems. Similarly, in the Nd‐system the complete pathway of crystallization resulting in hydroxylbastnasite was observed in the aragonite experiments, but no lanthanite formed in the dolomite experiments. On the other hand, experiments with Dy only resulted in tengerite and kozoite. This polymorph selection is a direct consequence of the ionic radii of the rare earth elements involved. The ionic radii of the La^3+^, Pr^3+^, Nd^3+^, and Dy^3+^ are 1.15, 1.09, 1.08, and 0.99 Å, respectively. Dy^3+^ ionic radius is the smallest among the examined REEs, and this size inhibits the crystallization of Dy‐lanthanite and Dy‐hydroxylbasnasite at the studied temperature‐pressure range. In this case, Dy‐tengerite is favored because its structure allows ions with sizes equal to or smaller than Nd^3+^.^[^ ^44^ ^]^

Also, the ionic radii of the REE^3+^ affect the unit cell dimensions of the REE‐carbonate isomorphs (**Table** **5**). Our experiments revealed that the largest unit cell parameters correspond to the lighter REE‐isomorphs, a consequence of the decrease in the ionic radii with the atomic weight of the REEs, also known as the lanthanide contraction. This agrees with the usual crystal chemistry of these minerals in nature and synthesis experiments. Lanthanite‐type isomorphs contain light REEs with larger ionic radii such as La, Ce, or Nd,^[^ ^36^, ^37^, ^45^, ^46^ ^]^ forming a mineral structure with two types of RE‐O polyhedra in 10‐fold and 8‐fold coordination. Similarly, hydroxylbastnasite isomorphs incorporate light REE (La, Ce, or Nd^[^ ^21^, ^36^, ^47^, ^48^, ^49^ ^]^) forming a hexagonal structure with the REE ion in 9‐fold coordinated with oxygen. However, at high‐pressure conditions, outside our experimental range, heavier REE can incorporate into the structure of hydroxylbastnasite.^[^ ^50^, ^51^, ^52^ ^]^ In between lanthanite and hydroxylbastnasite, kozoite isomorphs are intermediate phases, forming structures with 10‐fold REE‐O coordination, including lighter and heavier REEs (e.g., La, Nd, or Dy).^[^ ^36^ ^]^ It must be noted that kozoite‐type isomorphs with heavier REEs than Dy, results in different structures with smaller REE‐O coordination numbers, 8‐ and 9‐fold and orthorhombic or tetragonal crystal systems.^[^ ^48^ ^]^

**Table 5:** Unit cell parameters of original dolomite and aragonite used for the experiments and the La‐, Pr‐, Nd‐, and Dy‐bearing carbonate minerals crystallized in the experiments

Mineral	Space group	La‐system	Pr‐system	Nd‐system	Dy‐system
Dolomite	*R* $\bar{3}$ *a* = *b* = 4.8028(13) Å *c* = 15.9998(43) Å vol = 319.62(20) Å^3^				
Lanthanite	*Pbnb*	*a* = 9.5820(62) Å *b* = 17.0284(58) Å *c* = 9.0916(65) Å vol = 1482.6(15) Å^3^	*a* = 9.465(14) Å *b* = 16.941(10) Å *c* = 8.846(16) Å vol = 1418.5(34) Å^3^		
Kozoite	*Pnma*	*a* = 7.3983(41) Å *b* = 5.0342(28) Å *c* = 8.5818(49) Å vol = 319.62(31) Å^3^	*a* = 7.2633(28) Å *b* = 4.9787(18) Å *c* = 8.5120(33) Å vol = 307.81(20) Å^3^	*a* = 7.2107(31) Å *b* = 4.9459(21) Å *c* = 8.4718(36) Å vol = 302.13(22) Å^3^	*a* = 6.9795(18) Å *b* = 4.8302(12) Å *c* = 8.4427(21) Å vol = 284.63(12) Å^3^
Hydroxylbastnasite	*P* $\bar{6}$	*a* = 12.6176(28) Å *c* = 10.0225(24) Å vol = 1381.84(71) Å^3^	*a* = 12.4026(34) Å *c* = 9.9306(28) Å vol = 1322.91(81) Å^3^	*a* = 12.3372(75) Å *c* = 9.9094(64) Å vol = 1306.2(18) Å^3^	
Aragonite	*Pmcn* *a* = 4.9608(13) Å *b* = 7.9663(20) Å *c* = 5.7421(15) Å vol = 226.92(10) Å^3^				
Lanthanite	*Pbnb*	*a* = 9.5401(76) Å *b* = 17.0529(92) Å *c* = 8.9836(80) Å vol = 1461.5(19) Å^3^	*a* = 9.4504(54) Å *b* = 16.9250(78) Å *c* = 8.8922(46) Å vol = 1422.3(13) Å^3^	*a* = 9.4334(29) Å *b* = 16.8818(50) Å *c* = 8.8687(27) Å vol = 1412.37(75) Å^3^	
Kozoite	*Pnma*	*a* = 7.4182(57) Å *b* = 5.0481(39) Å *c* = 8.5980(68) Å vol = 321.97(44) Å^3^	*a* = 7.2595(33) Å *b* = 4.9719(23) Å *c* = 8.5064(39) Å vol = 307.03(24) Å^3^	*a* = 7.2077(31) Å *b* = 4.9063(21) Å *c* = 8.4786(38) Å vol = 299.83(23) Å^3^	*a* = 6.9928(47) Å *b* = 4.8461(33) Å *c* = 8.4543(57) Å vol = 286.50(34) Å^3^
Hydroxylbastnasite	*P* $\bar{6}$	*a* = 12.6128(99) Å *c* = 10.0283(80) Å vol = 1381.6(24) Å^3^	*a* = 12.4071(51) Å *c* = 9.9354(43) Å vol = 1324.5(12) Å^3^	*a* = 12.3541(38) Å *c* = 9.8958(39) Å vol = 1307.99(96) Å^3^	
Tengerite	*Bb*21*m*				*a* = 6.0796(42) Å *b* = 9.1918(76) Å *c* = 15.136(11) Å vol = 845.9(11) Å^3^

## 4 Conclusions

The reaction between dolomite/aragonite seeds and REE‐bearing solutions (La, Pr, Nd, Dy) at low hydrothermal conditions (21–220 °C) resulted in the pseudomorphic mineral replacement of the host grains by REE‐carbonates, periphery inward. These newly formed REE carbonates follow a transformation from lanthanite (or tengerite, only in the Dy‐doped aragonite system), via kozoite to hydroxylbasnasite. The crystallization pathways are driven by the ionic radius of the rare earth element in question, the temperature and the solubility and the dissolution rate of the host minerals. These results provide fundamentals on the formation of REE‐carbonates and on the exact crystallization kinetics. Hence our study provides primary information that can be applied in a wide range of areas, which includes but is not limited to innovative material design, REE separation, exploration, exploitation and recycling methods.

## 5 Experimental Section

The general experimental method was set up by adding 0.1 g dolomite or 0.1 g aragonite with the size of 0.5–1.0 mm to 50 mL of 50 × 10^−3^ m rare earth bearing aqueous (Milli‐Q) solutions (pH ≈ 5.1). The solutions each were prepared with single rare earth elements using La(NO_3_)_3_ · 6H_2_O, Pr(NO_3_)_3_ · 6H_2_O, Nd(NO_3_)_3_ · 6H_2_O, and Dy(NO_3_)_3_ · 6H_2_O reagents (Sigma‐Aldrich; 99.99% trace metals basis). The solutions and solids were placed in 50 mL Teflon‐lined stainless‐steel autoclaves at different temperatures (21, 50, 80, 165, and 220 °C) and saturated water vapor pressures. Solid samples were taken carefully at increasing time intervals which were then placed into an oven at 50 °C for 30 min to remove any excess water.

The dried samples were first examined by a binocular microscope (ZEISS SteREO Discovery.V8 with an 8:1 manual zoom range; 6.3–50.4×) to reveal any major change in the morphology and/or the color of the crystals. Following, the samples were ground and analyzed with powder X‐ray diffraction (XRD) to identify and quantify any newly formed crystalline compounds. XRD analyses were carried out using a Bruker D5000 XRD (Cu Kα radiation, 0.01° step^−1^ from 5 to 70° 2θ at 1° min^−1^). The XRD patterns were identified with Diffract Suite EVA software from Bruker in combination with the Powder Data File (PDF‐4, The International Centre for Diffraction Data). Rietveld refinement software (TOPAS)^[^ ^53^ ^]^ was used to conduct pattern‐matching refinement, quantification of the crystalline phases; in all cases, the quantitative XRD errors were lower than 1–2 wt%, no further pre‐processing of data (normalization, etc.) was needed. The quantification of the reaction extents (fraction transformed) was used to provide information on the rate of transformation with the empirical *Avrami* equation^[^ ^54^ ^]^

(1) $$\begin{array}{c} y=1-\exp{\left(-kt\right)}^{n} \end{array}$$

where *k* is a rate constant (rate of transformation), *t* is time, *y* is the fraction transformed, and *n* is a constant which depends on the transition mechanism. Rewriting the *Avrami* Equation (1) gives Equation (2)

(2) $$\begin{array}{c} -\ln\ln\left(1-y\right)=n\ln k+n\ln t \end{array}$$

The reaction with kinetics that conform to this equation give a straight line when −ln ln (1 − *y*) is plotted against ln *t*.^[^ ^55^, ^56^ ^]^ The empirical parameter *n* value is given by the value of the slope, which is used to compare reaction mechanism. Parallel lines indicate a constant value of *n*, suggesting that the reaction mechanism is the same. The intercept on the *y* axis gives the value of *n* ln *k*, by which the *k* value can be determined.

Scanning electron microscopy (SEM) was used to characterize the changes in the morphology of the host mineral (dolomite or aragonite) and of the newly formed crystalline phases. SEM was also used to carry out elemental analysis on areas of interest by using energy dispersive spectroscopy (EDS) analysis. All samples were carbon‐coated and placed into a Tescan S8000 FEG‐SEM operating under high vacuum conditions and equipped with four Oxford Instruments NanoAnalysis X‐Max 170 mm^2^ EDS detector running Oxford Instruments NanoAnalysis AZtecTimed analysis software. All analyses were performed using a beam current of 300 pA and an accelerating voltage of either 5 kV, for detailed imaging, or 20 kV, for EDX analysis.

The saturation indices of REE‐bearing carbonates during the equilibration of the REE‐bearing aqueous solutions with respect to dolomite or aragonite were calculated with the hydrogeochemical code PHREEQC^[^ ^57^ ^]^ using the LLNL database^[^ ^58^ ^]^ and the solubility products of REE‐bearing carbonates determined by Essington and Mattigod^[^ ^24^ ^]^ and Voigt et al.^[^ ^26^ ^]^

Data visualization and statistical information were retrieved by the visual data tool, DataGraph (version 4.71).^[^ ^59^ ^]^ Statistical values are summarized in Table S3 (Supporting Information).

## Conflict of Interest

The authors declare no conflict of interest.

## Supporting information

Supporting Information

## Data Availability

The data that support the findings of this study are available from the corresponding author upon reasonable request.
