# Supplementary material for: Targeted Crystallization of Rare Earth Carbonate Polymorphs at Hydrothermal Conditions via Mineral Replacement Reactions
Source: Glob Chall. 2022 Sep 19;7(2):2200085. doi: 10.1002/gch2.202200085 (PMC9900722; doi:10.1002/gch2.202200085)
Supplement: Supplementary file 1 — Supporting Information [file GCH2-7-2200085-s001.pdf]

Global Challenges
Open Access

Supporting Information

for *Global Challenges,* DOI: 10.1002/gch2.202200085

Targeted Crystallization of Rare Earth Carbonate Polymorphs at Hydrothermal Conditions via Mineral Replacement Reactions

Adrienn Maria Szucs, Melanie Maddin, Daniel Brien, Paul Christopher Guyett, and Juan Diego Rodriguez-Blanco**

Supporting Information

Targeted Crystallization of Rare Earth Carbonate Polymorphs at Hydrothermal Conditions via Mineral Replacement Reactions.

Adrienn Maria Szucs, Melanie Maddin, Daniel Brien, Paul Christopher Guyett, Juan Diego Rodriguez-Blanco**

Table SI-1. Ca and Mg content (atomic %) of REE-bearing carbonates in dolomite experiments determined with energy dispersive spectroscopy (EDS) analysis using scanning electron microscopy (SEM). BLD = below detection limit.

**REE-carbonate and divalent cation**		**Temperature (°C)**				
		21	50	80	165	220
La-lanthanite [$La_2(CO_3)_3 \cdot 8H_2O$]	Ca^{2+}	BLD	0.16 ± 0.10	BLD	—	—
	Mg^{2+}	BLD	BLD	BLD	—	—
Pr-lanthanite [$Pr_2(CO_3)_3 \cdot 8H_2O$]	Ca^{2+}	0.08 ± 0.09	—	—	—	—
	Mg^{2+}	BLD	—	—	—	—
Nd-lanthanite [$Nd_2(CO_3)_3 \cdot 8H_2O$]	Ca^{2+}	—	—	—	—	—
	Mg^{2+}	—	—	—	—	—
La-kozoite [$LaCO_3(OH)$]	Ca^{2+}	—	0.16 ± 0.10	BLD	—	—
	Mg^{2+}	—	BLD	BLD	—	—
Pr-kozoite [$PrCO_3(OH)$]	Ca^{2+}	0.27 ± 0.10	0.10 ± 0.12	0.11 ± 0.06	BLD	0.20 ± 0.04
	Mg^{2+}	0.08 ± 0.19	BLD	BLD	BLD	0.07 ± 0.17
Nd-kozoite [$NdCO_3(OH)$]	Ca^{2+}	0.25 ± 0.13	0.31 ± 0.04	0.08 ± 0.09	BLD	BLD
	Mg^{2+}	0.21 ± 0.24	BLD	0.06 ± 0.17	BLD	BLD
Dy-kozoite [$DyCO_3(OH)$]	Ca^{2+}	—	—	0.07 ± 0.09	BLD	BLD
	Mg^{2+}	—	—	0.97 ± 0.14	0.87 ± 0.13	1.06 ± 0.17
La-hydroxylbastnasite [$LaCO_3(OH)$]	Ca^{2+}	—	—	—	BLD	BLD
	Mg^{2+}	—	—	—	BLD	BLD
Pr-hydroxylbastnasite [$PrCO_3(OH)$]	Ca^{2+}	—	—	—	BLD	BLD
	Mg^{2+}	—	—	—	BLD	BLD
Nd-hydroxylbastnasite [$NdCO_3(OH)$]	Ca^{2+}	—	—	—	0.04 ± 0.07	BLD
	Mg^{2+}	—	—	—	0.12 ± 0.19	BLD

Table SI-2. Ca content (atomic %) of REE-bearing carbonates in aragonite experiments determined with energy dispersive spectroscopy (EDS) analysis using scanning electron microscopy (SEM). BLD = below detection limit.

**Temperature °C**	21	50	80	165	220
La-lanthanite [$La_2(CO_3)_3{\cdot}8H_2O$]	0.15 ± 0.04	1.39 ± 1.01	—	—	—
Pr-lanthanite [$Pr_2(CO_3)_3{\cdot}8H_2O$]	1.00 ± 0.047	1.40 ± 0.13	—	—	—
Nd-lanthanite [$Nd_2(CO_3)_3{\cdot}8H_2O$]	0.32 ± 0.19	—	—	—	—
La-kozoite [$LaCO_3(OH)$]	—	0.36 ± 0.07	1.22 ± 0.74		—
Pr-kozoite [$PrCO_3(OH)$]	0.32 ± 0.05	2.57 ± 2.57	BLD	BLD	BLD
Nd-kozoite [$NdCO_3(OH)$]	1.27 ± 0.34	0.29 ± 0.04	BLD	BLD	BLD
Dy-kozoite [$DyCO_3(OH)$]	0.25 ± 0.05	—	0.10 ± 0.08	0.42 ± 0.59	0.04 ± 0.10
La-hydroxylbastnasite[$LaCO_3(OH)$]	—	—	—	0.12 ± 0.17	BLD
Pr-hydroxylbastnasite[$PrCO_3(OH)$]	—	—	—	BLD	BLD
Nd-hydroxylbastnasite[$NdCO_3(OH)$]	—	—	—	BLD	BLD
Dy-tengerite [$Dy_2(CO_3)_3{\cdot}2\text{-}3H_2O$]	—	—	0.14 ± 0.09	—	—

Table SI-3. Statistical values of the fitted linear regression for all the experiments.

Experiment	Sample Size	Slope	Standard Error (Slope)	Y Intercept	Standard Error Y Intercept	X Intercept	R^2	RMSE (Root mean square error)	Linear Regression Equation
Dol-La-21C	4	0.97001	0.096745	-16.738	1.4327	17.255	0.98049	0.10074	(-16.738) + 0.97001*x
Dol-La-50C	6	1.5127	0.1911	-21.23	2.6004	14.035	0.93999	0.63151	(-21.23) + 1.5127*x
Dol-La-80C	4	1.0902	0.59012	-14.592	7.9099	13.384	0.63053	1.4675	(-14.592) + 1.0902*x
Dol-La-165C	3	2.3627	0.48336	-30.548	6.4172	12.929	0.95983	0.52738	(-30.548) + 2.3627*x
Dol-La-220C	2	1.7305	–	-18.792	–	10.859	1	–	(-18.792) + 1.7305*x
Dol-Pr-21C	4	1.212	0.071453	-20.839	1.0582	17.195	0.9931	0.074401	(-20.839) + 1.212*x
Dol-Pr-50C	5	1.0482	0.18033	-13.942	2.4392	13.301	0.91844	0.58877	(-13.942) + 1.0482*x
Dol-Pr-80C	4	1.1082	0.49932	-14.557	6.6928	13.136	0.71121	1.2417	(-14.557) + 1.1082*x
Dol-Pr-165C	3	3.1345	0.09532	-41.168	1.2655	13.134	0.99908	0.104	(-41.168) + 3.1345*x
Dol-Pr-220C	2	2.1528	–	-24.056	–	11.174	1	–	(-24.056) + 2.1528*x
Dol-Nd-21C	3	1.9916	0.19751	-34.602	2.976	17.375	0.99026	0.097268	(-34.602) + 1.9916*x
Dol-Nd-50C	5	1.6597	0.52926	-24.114	7.4162	14.529	0.76625	1.2128	(-24.114) + 1.6597*x
Dol-Nd-80C	5	1.1398	0.20591	-15.47	2.8465	13.572	0.91083	0.63576	(-15.47) + 1.1398*x
Dol-Nd-165C	4	2.233	0.50271	-30.275	6.8599	13.558	0.90796	0.83223	(-30.275) + 2.233*x
Dol-Nd-220C	2	2.864	–	-32.921	–	11.495	1	–	(-32.921) + 2.864*x
Dol-Dy-21C	–	–	–	–	–	–	–	–	–
Dol-Dy-50C	–	–	–	–	–	–	–	–	–
Dol-Dy-80C	4	2.128	0.81736	-30.953	11.748	14.545	0.77217	1.2668	(-30.953) + 2.128*x

Dol-Dy-165C	4	1.6372	0.69832	-22.215	9.5291	13.569	0.73321	1.1561	(-22.215) + 1.6372*x
Dol-Dy-220C	2	3.7074	–	-43.434	–	11.716	1	–	(-43.434) + 3.7074*x
Ara-La-21C	4	0.49673	0.04502	-9.9338	0.66672	19.998	0.98384	0.046877	(-9.9338) + 0.49673*x
Ara-La-50C	6	0.75205	0.13934	-9.455	1.8961	12.572	0.87926	0.46047	(-9.455) + 0.75205*x
Ara-La-80C	4	0.84709	0.39266	-10.59	5.2631	12.501	0.69943	0.97647	(-10.59) + 0.84709*x
Ara-La-165C	3	2.3252	0.74137	-30.149	9.8426	12.966	0.90772	0.80889	(-30.149) + 2.3252*x
Ara-La-220C	2	2.3107	–	-26.024	–	11.262	1	–	(-26.024) + 2.3107*x
Ara-Pr-21C	3	0.43181	0.12264	-7.8904	1.8041	18.273	0.92535	0.12022	(-7.8904) + 0.43181*x
Ara-Pr-50C	6	0.90963	0.22249	-12.216	3.0275	13.429	0.8069	0.73524	(-12.216) + 0.90963*x
Ara-Pr-80C	4	0.84389	0.3011	-10.338	4.0359	12.25	0.79706	0.74879	(-10.338) + 0.84389*x
Ara-Pr-165C	3	2.0139	0.49575	-25.669	6.5817	12.746	0.94287	0.5409	(-25.669) + 2.0139*x
Ara-Pr-220C	2	2.864	–	-32.921	–	11.495	1	–	(-32.921) + 2.864*x
Ara-Nd-21C	4	1.5201	0.1308	-25.128	1.9371	16.531	0.98541	0.13619	(-25.128) + 1.5201*x
Ara-Nd-50C	5	1.1383	0.1477	-15.311	2.0184	13.45	0.95192	0.48669	(-15.311) + 1.1383*x
Ara-Nd-80C	5	0.82849	0.39232	-11.457	5.4236	13.829	0.59783	1.2114	(-11.457) + 0.82849*x
Ara-Nd-165C	3	1.794	0.90021	-22.787	11.951	12.702	0.79885	0.98219	(-22.787) + 1.794*x
Ara-Nd-220C	2	2.864	–	-32.921	–	11.495	1	–	(-32.921) + 2.864*x
Ara-Dy-21C	–	–	–	–	–	–	–	–	–
Ara-Dy-50C	–	–	–	–	–	–	–	–	–
Ara-Dy-80C	4	1.4122	0.27656	-19.265	3.975	13.641	0.92876	0.42864	(-19.265) + 1.4122*x
Ara-Dy-165C	3	1.7397	0.88385	-22.018	11.734	12.656	0.79484	0.96435	(-22.018) + 1.7397*x
Ara-Dy-220C	2	2.1253	–	-23.712	–	11.157	1	–	(-23.712) + 2.1253*x

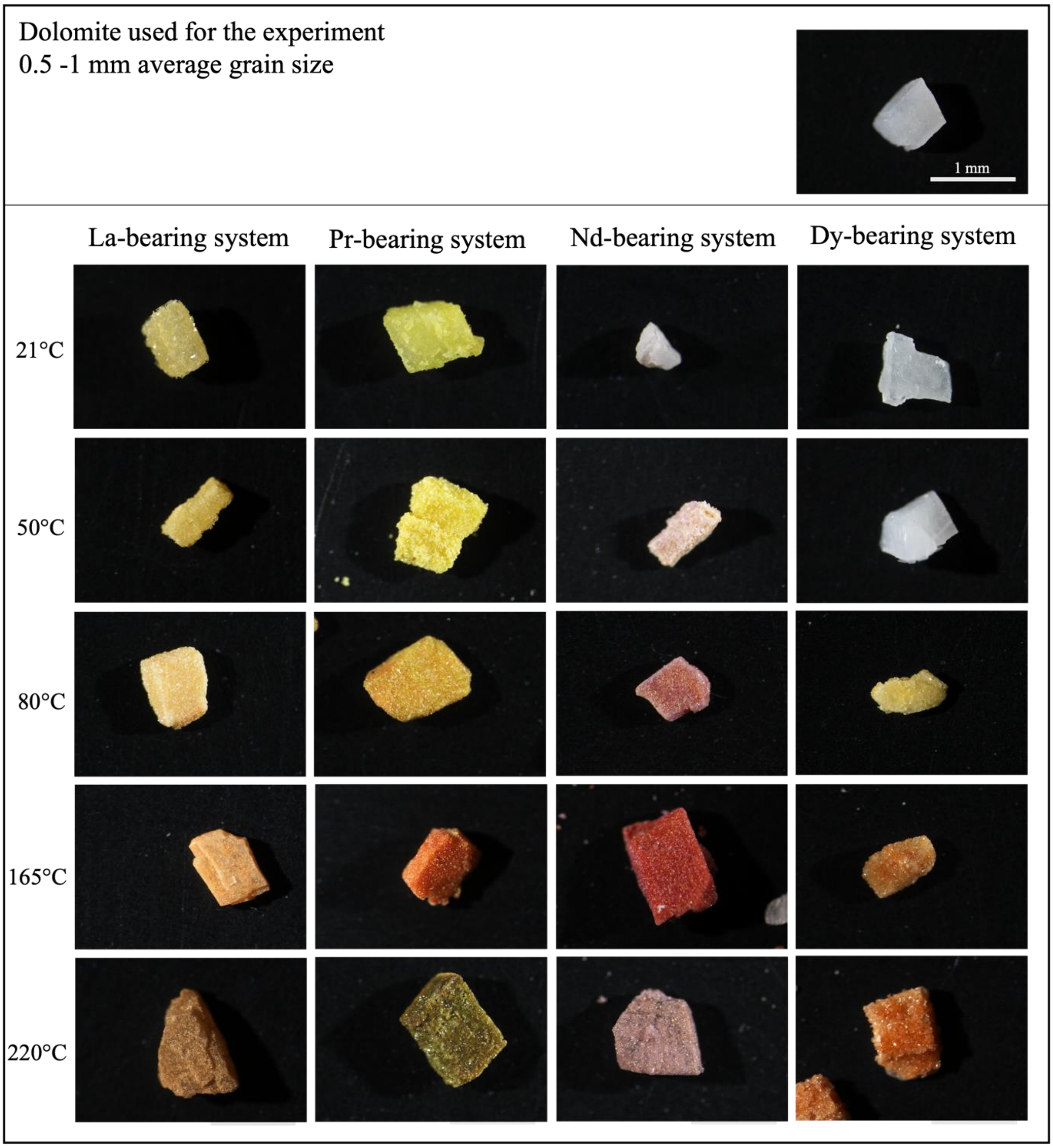

Figure S1. Binocular microscope images of the final solids obtained after the interaction of dolomite with REE-bearing solutions, showing the changes in the color and opacity.

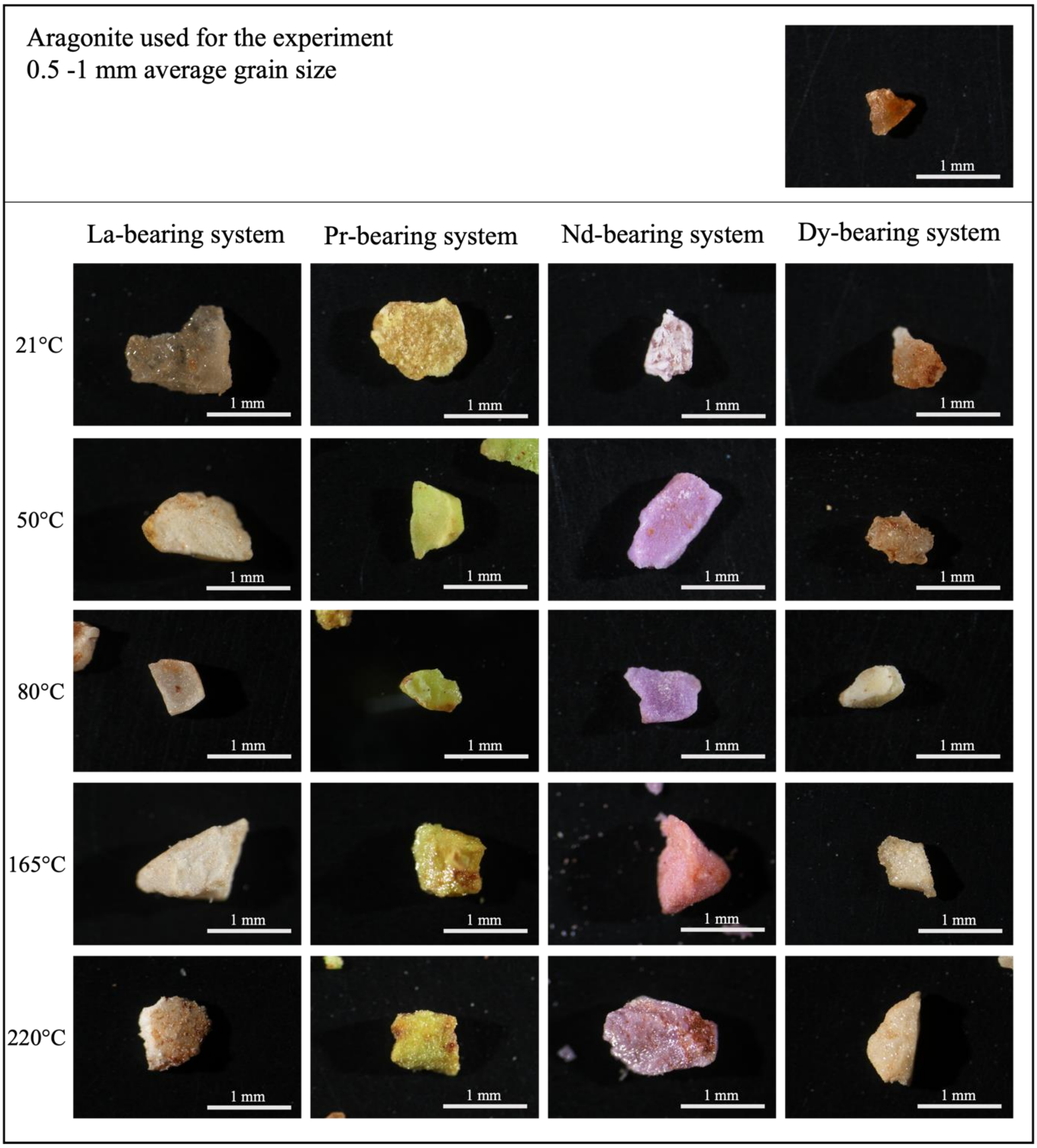

Figure S2. Binocular microscope images of the final solids obtained after the interaction of aragonite with REE-bearing solutions, showing the changes in the color and opacity.

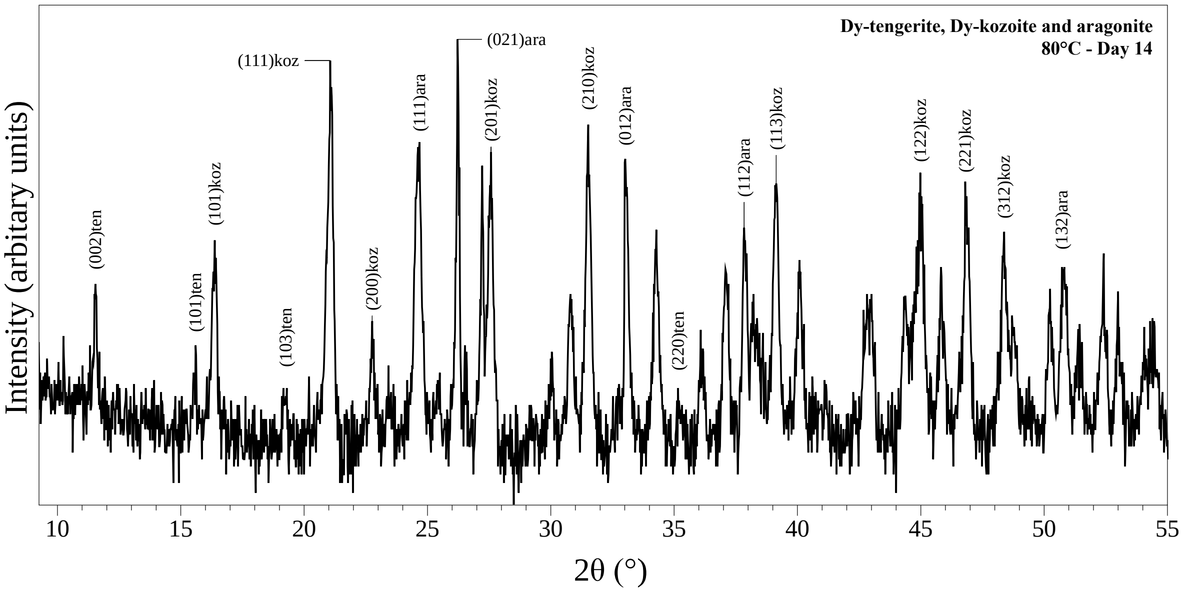

Figure S3. Powder XRD pattern of Dy-tengerite in the presence of Dy-kozoite and aragonite obtained as the result of the experiment at 80 °C on day 14.

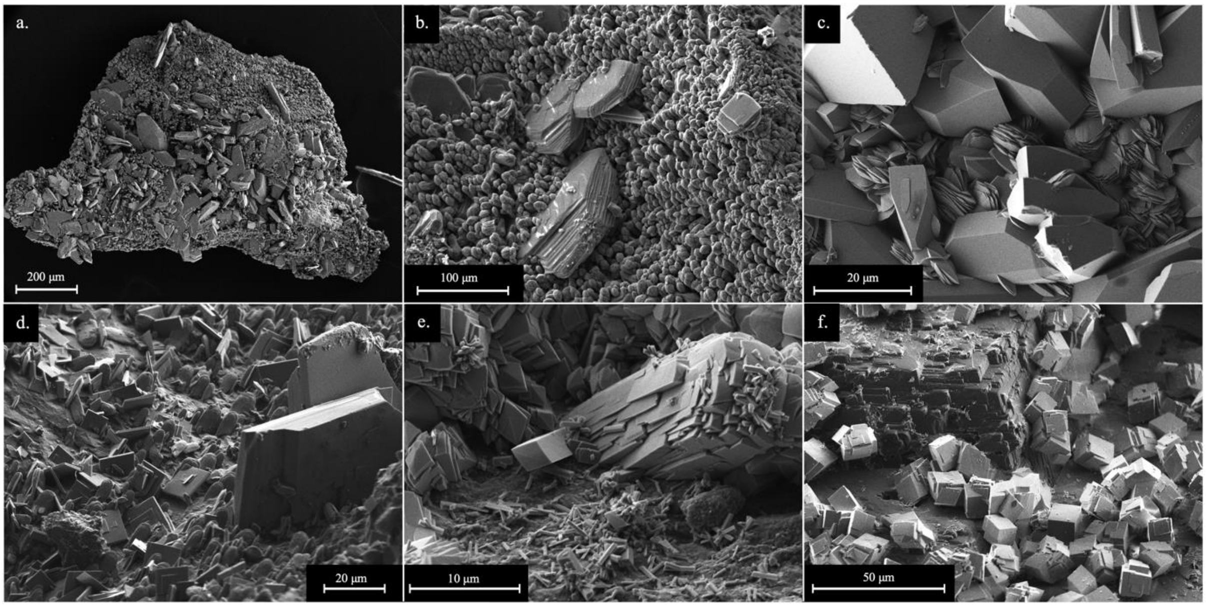

Figure S4. SEM images of (a) an aragonite grain fully covered by La-lanthanite and La-kozoite, (b) morphology of the thin platy crystals of La-lanthanite and elongated prisms of La-kozoite, (c) Nd-hydroxylbastnasite forming in between Nd-kozoite, (d) La-lanthanite forming on the surface of aragonite alongside with La-kozoite, (e) La-kozoite forming on the surface of aragonite, (f) Dy-kozoite prisms forming on the surface of dolomite.

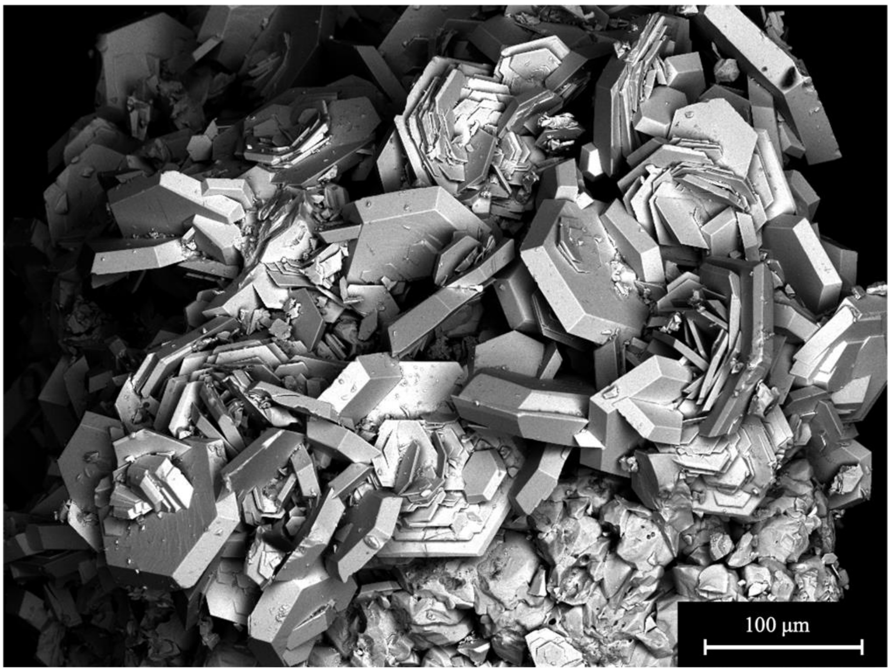

Figure S5. SEM image of Dy-kozoite with hexagonal morphology forming on the surface of dolomite after 3 days at 220 °C.

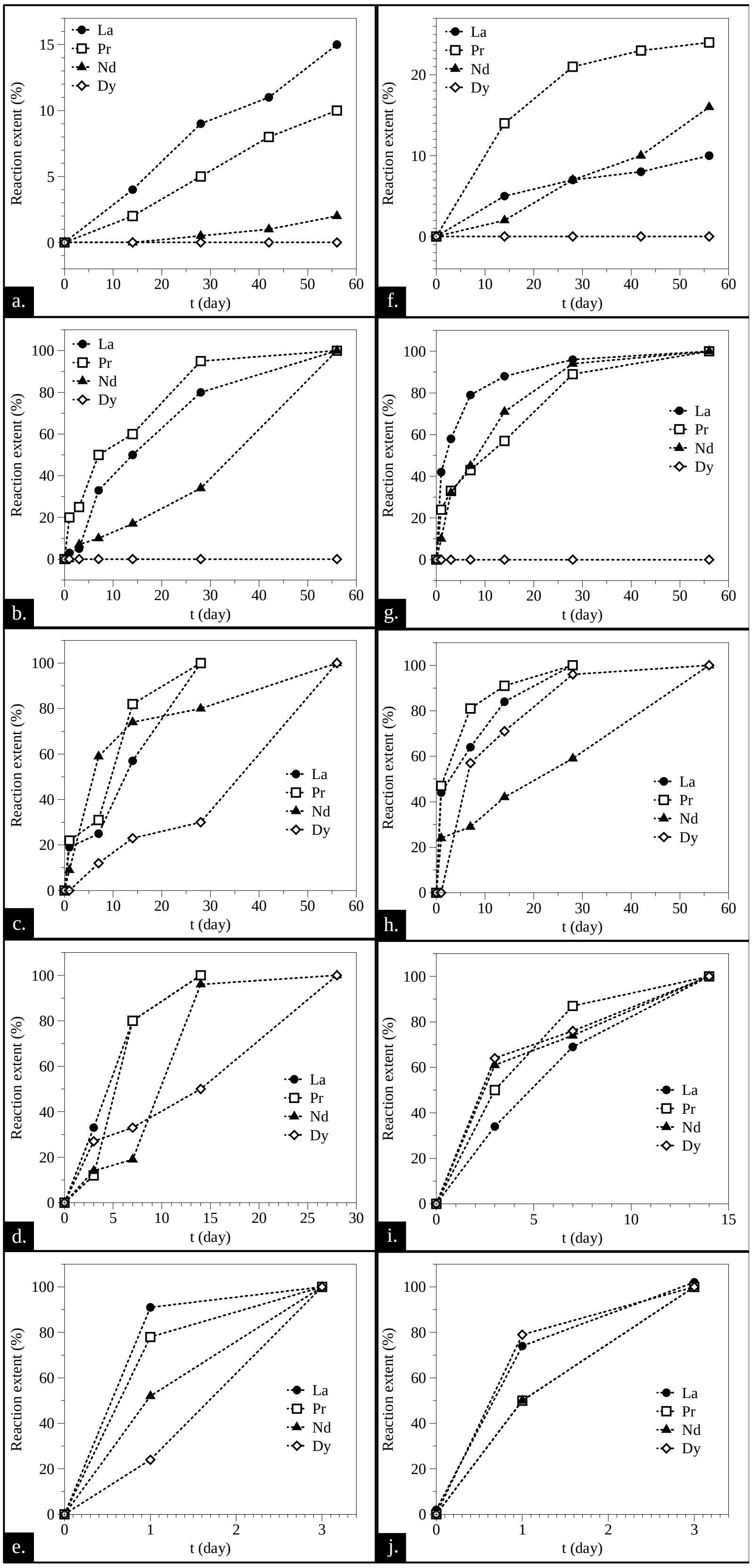

Figure S6. Reaction extents plotted against time showing the effect of single REEs on the reaction with dolomite (a-e) and aragonite (f-j) at 21, 50, 80, 165 and 220 °C (top to bottom).
